# Supplementary material for: Tuning the dynamic range of bacterial promoters regulated by ligand-inducible transcription factors
Source: Nat Commun. 2018 Jan 4;9:64. doi: 10.1038/s41467-017-02473-5 (PMC5754348; doi:10.1038/s41467-017-02473-5)
Supplement: Supplementary file 1 — Supplementary Information [file 41467_2017_2473_MOESM1_ESM.pdf]

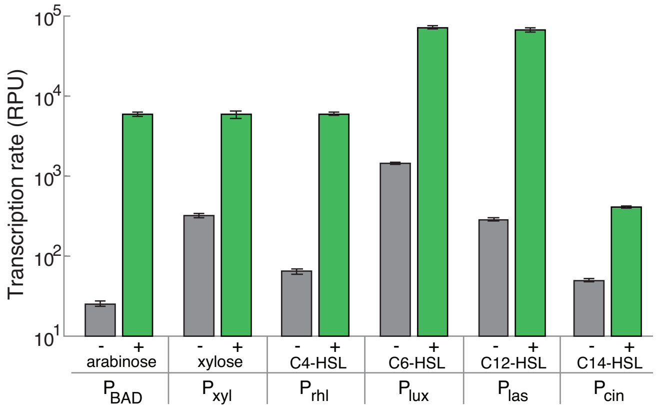

Supplementary Figure 1. Ligand-inducible promoters have different dynamic ranges. Experimentally measured transcription rates of 6 ligand-inducible promoters (P_{BAD}, P_{xyl}, P_{rhl}, P_{lux}, P_{las}, and P_{cin}) with and without saturating concentrations of their respective inducers (5 mM arabinose, 5 mM xylose, 10 μM C4-HSL, 0.1 μM 3O-C6-HSL, 0.1 μM 3O-C12-HSL, and 1 μM 3OH-C14-HSL, respectively). Error bars represent the standard deviation calculated from experiments performed with biological triplicates.

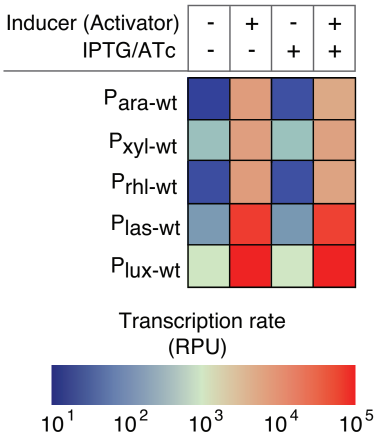

Supplementary Figure 2. Transcription rates of wild-type promoters containing operator sites for one of five different transcriptional activators (AraC, XylR, RhlR, LasR, or LuxR). These negative controls confirm that the AND gate behavior observed in Figure 5 is attributable to the hybrid promoters.

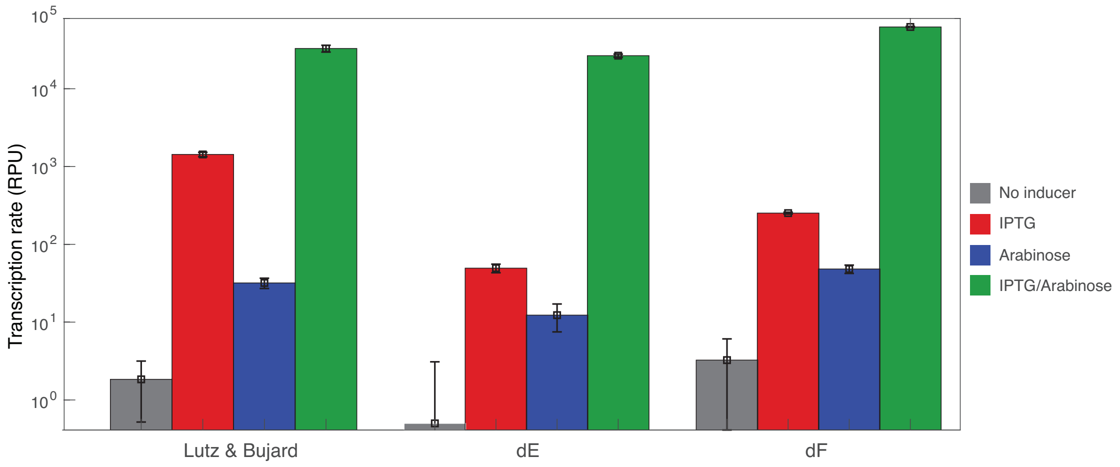

Supplementary Figure 3. Comparison of leakiness of the dE and dF combinations of the hybrid $P_{ara/lac}$ promoter to the same promoter reported in reference 13 of the main text (Lutz and Bujard, 1997). The dE and dF combinations developed in this study exhibit lower leakiness than the Lux & Bujard promoter, particularly when only IPTG is added.

Supplementary Table 1. Strains used in the promoter study

Strain Name	Genotype
MG1655	*E. coli* K12 MG1655 (wild type)
CY011	MG1655 *ΔlacI*
CY013	MG1655 *ΔlacI ΔsdiA*
CY015	MG1655 *ΔlacI ΔsdiA* + P_{wt}-*araC*, P_{trc*}-*rhlR*, P_{Iq}-*lasR*
CY019	MG1655 *ΔlacI ΔsdiA*+ P_{Iq}-*lacI*, P_{N25}-*tetR*
CY021	MG1655 *ΔlacI ΔsdiA* + P_{Iq}-*lacI*, P_{N25}-*tetR*, P_{wt}-*araC*, P_{trc*}-*rhlR*, P_{Iq}-*lasR*
CY027	BW25113 *ΔlacI ΔaraC ΔsdiA* + P_{trc*}-*rhlR*, P_{trc*}-*cinR*

Supplementary Table 2. General plasmids used in the promoter study

Plasmid	Open Reading Frame	Origin/Resistance	Note
pCH1	flt-Cm^R-flt	pSC101, Cm^R	Gene KO
pCH35	P_{wt}-*araC*, P_{trc*}-*rhlR*, P_{Iq}-*lasR*	pSC101, Cm^R	*araC, rhlR, lasR* KI
pCH104	P_{Iq}-*cinR*	pSC101, Cm^R	*cinR* carrier
pCH161†	P_{Iq}-*lacI*, P_{N25}-*tetR*	pSC101, Cm^R	*lacI*, *tetR* KI
pCH192	P_{Iq}-*luxR*	pSC101, Cm^R	*luxR* carrier
pCHxxx‡	P_{xxx}-*BCD2*sYFP*	pMB1+ROP, Kan^R	Promoters

† pCH161 is a derivative of pZA24 described in reference 13 of the main text (Lutz and Bujard, 1997).

‡ This is the batch of reporter plasmids with the engineered promoters described in more detail in Tables S4 and S5.

Supplementary Table 3. Elements in promoter construction

Motifs	Name	Sequence
Activator	AraI_1+I_2	catagcatttttatccataagattagcggatcctacct
(Upstream to	XylI_1+I_2	gaaataaaccaaaaatcgtaatcgaaagataaaaatctgtaa
-36)	RhlO	tcctgtgaaatctggcagt
	LasO	aatctatctcatttgctagt
	LuxO	acctgtaggatcgtacagt
	CinO	(ga)gggggcctatctgagggaa
Repressor	LacO_s	Ttgtgagcgctcacaatt
(Spacer, -29 to -13)	LacO_1	ttgtgagcggataacaa
	TetO_2	tccctatcagtgatagaGA
Repressor	LacO_1	aattgtgagcggataacaatt
(~-400)	TetO_2	tccctatcagtgatagaga
-6 to +1		gagcaca

Capitalized base pairs overlap with either the -35 site or the -10 site. Underlined base pairs correspond to the +1 site. The AND promoters contain a distal repressor site + 400bp spacer + activator binding site + -35 site (TTTACT) + spacer (17bp repressor binding site) + -10 site (“D” or “G” see Fig. 2a) + GAGCACA + repressor binding site.

Supplementary Table 4. Table of binding energy values (E/k_BT) obtained from thermodynamic fits

-35	Sequence	E/k_BT AraC	E/k_BT AraC/LacI	E/k_BT LasR	E/k_BT LasR/LacI
a	CCCGGG	7.88	7.17	9.95	9.48
b	CTGACA	6.51	6.4	7.31	6.89
c	TTGTGA	4.64	4.86	5.25	5.04
d	TTTACA	2.67	2.94	3.90	3.69
e	TAGACA	3.29	3.28	3.45	3.32
f	TTGACA	0	0	0	0

-10	Sequence	E/k_BT AraC	E/k_BT AraC/LacI	E/k_BT LasR	E/k_BT LasR/LacI
A	CCAGTC	13.4	11.58	12.44	12.58
B	TATGTT	4.79	4.6	4.92	4.7
C	TACTGT	4.92	4.57	4.99	4.82
D	TAAATT	4.38	4.21	4.65	4.61
E	GATACT	3.74	3.54	4.41	4.28
F	GATAAT	3	2.63	3.48	3.37
G	TATAGT	1.7	1.93	1.98	2.02
H	TATAAT	0	0	0	0

Values are provided as E/k_BT because $-\ln(K_{eq}) = E/k_BT$. In each fit, simply add each of the -35 and -10 deconvolved $\ln(K_{eq})$ value to calculate the composite $\ln(K_{eq})$ value for each of the 48 combinations. Values are relative to sequences f and H.

Supplementary Table 5. Addgene identifiers for deposited strains and plasmids

Item	Descriptor	Identity	Addgene
CY15	MG1655 *ΔlacI ΔsdiA* + P_{wt}-*araC*, P_{trc*}-*rhlR*, P_{Iq}-*lasR*	Strain	104961
CY19	MG1655 *ΔlacI ΔsdiA*+ P_{Iq}-*lacI*, P_{N25}-*tetR*	Strain	104864
CY21	MG1655 *ΔlacI ΔsdiA* + P_{Iq}-*lacI*, P_{N25}-*tetR*, P_{wt}-*araC*, P_{trc*}-*rhlR*, P_{Iq}-*lasR*	Strain	104865
CH9	Para-Reference Standard	Strain	104866
CH192	LuxR helper plasmid to be used with CY19	Plasmid	104960
CH42	Plas-eG	Plasmid	104867
CH110	Para-eG	Plasmid	104868
PE16	Para/lac-eG	Plasmid	104869
PE17	Plas/lac-eG	Plasmid	104870
PE18	Plux/lac-eG	Plasmid	104871
PE19	Prhl/lac-eG	Plasmid	104872
PE20	Pxyl/lac-eG	Plasmid	104881
PE21	Para/tet-eG	Plasmid	104882
PE22	Plas/tet-eG	Plasmid	104883
PE23	Plux/tet-eG	Plasmid	104884
PE24	Prhl/tet-eG	Plasmid	104885
PE25	Pxyl/tet-eG	Plasmid	104886
PE26	Para/lac/tet-eG	Plasmid	104887
PE27	Plas/lac/tet-eG	Plasmid	104888
PE28	Plux/lac/tet-eG	Plasmid	104889
PE29	Prhl/lac/tet-eG	Plasmid	104890
PE30	Pxyl/lac/tet-eG	Plasmid	104891

Supplementary Note 1: Mathematical Modeling

Mathematical model with a single transcription factor

We start with the case of a single transcription factor (activator) only. We define all energies relative to some ground state. In this case we have four possible promoter binding states: 00, $A0$, 0σ, and $A\sigma$, corresponding to the unoccupied state, activator or σ-factor only, and activator and σ-factor both bound to DNA. This gives us the following energies, all of which are measured relative to that of the unoccupied state, 00,

$$\Delta G_{A0} = -\ln(KH^{+}(I)) \tag{1}$$
$$\Delta G_{0\sigma} = \Delta G_{-10,i} + \Delta G_{-35,j} \tag{2}$$
$$\Delta G_{A\sigma} = \Delta G_{A0} + \Delta G_{0\sigma} + \beta_{A\sigma}. \tag{3}$$

Here $\Delta G_{-10,i}$ and $\Delta G_{-35,j}$ correspond to the changes in the binding energy due to changes in sequences in the vicinity of the -10 and -35 sites. The subscripts i and j correspond to different promoters in the library. The constant $\beta_{A\sigma}$ is the drop in energy for binding the σ-factor when the activator is bound. To model the effect of inducer, we introduce an increasing Hill function of the form $H^{+}(I) = I^n/(C^n + I^n)$, where I denotes inducer concentration. This models the fraction of activator bound by the inducer, and hence the expected affinity of the activator for DNA. At full induction we have $\Delta G_{A0} = -\ln K$, although this is not important for the following derivation. Note that each ΔG is relative to $k_B T$, where k_B is Boltzmann's constant and T is the temperature.

Given these energies, the probability of σ-factor being bound is

$$P(\sigma) = \frac{e^{-\Delta G_{0\sigma}} + e^{-\Delta G_{A\sigma}}}{1 + e^{-\Delta G_{A0}} + e^{-\Delta G_{0\sigma}} + e^{-\Delta G_{A\sigma}}}, \tag{4}$$

where we have used $e^{-\Delta G_{00}} = 1$, since $\Delta G_{00} = 0$.

Without inducer, we assume that the probability of activator binding is negligible. We therefore write the probability $P_{-}(\sigma)$ of σ-factor binding in the absence of inducer as

$$P_{-}(\sigma) \approx \frac{e^{-\Delta G_{0\sigma}}}{1 + e^{-\Delta G_{0\sigma}}} = \frac{e^{-(\Delta G_{-10,i} + \Delta G_{-35,j})}}{1 + e^{-(\Delta G_{-10,i} + \Delta G_{-35,j})}}. \tag{5}$$

We assume that in the presence of inducer, the probability of activator and σ-factor both binding DNA is much higher than the probability of σ-factor alone binding, so that $\exp(-\Delta G_{A\sigma}) \gg \exp(-\Delta G_{0\sigma})$. Using Eqs.(2–3), we therefore have $\exp(-\Delta G_{A0} - \beta_{A\sigma}) \gg 1$. We write the probability that σ-factor binds at full induction as

$$\begin{aligned} P_{+}(\sigma) &\approx \frac{e^{-\Delta G_{A\sigma}}}{1 + e^{-\Delta G_{A0}} + e^{-\Delta G_{A\sigma}}} \\ &= \frac{e^{-(\Delta G_{A0} + \beta_{A\sigma} + \Delta G_{-10,i} + \Delta G_{-35,j})}}{1 + e^{-\Delta G_{A0}} + e^{-(\Delta G_{A0} + \beta_{A\sigma} + \Delta G_{-10,i} + \Delta G_{-35,j})}}. \end{aligned}$$

After multiplying the numerator and denominator by $\exp(-\gamma)$ where $\gamma = \ln(1 + e^{-\Delta G_{A0}})$, we obtain

$$P_{+}(\sigma) \approx \frac{e^{-(\gamma + \Delta G_{A0} + \beta_{A\sigma} + \Delta G_{-10,i} + \Delta G_{-35,j})}}{1 + e^{-(\gamma + \Delta G_{A0} + \beta_{A\sigma} + \Delta G_{-10,i} + \Delta G_{-35,j})}} = \frac{e^{-(\gamma_{+} + \Delta G_{-10,i} + \Delta G_{-35,j})}}{1 + e^{-(\gamma_{+} + \Delta G_{-10,i} + \Delta G_{-35,j})}}, \tag{6}$$

where $\gamma_{+} = \gamma + \Delta G_{A0} + \beta_{A\sigma}$.

Importantly, Eq. (5) and Eq. (6) have the same form, and differ in the single parameter γ_{+}.

The transcription rates are therefore approximately given by

$$TR_{-}(i,j) = \alpha \frac{e^{-(\Delta G_{-10,i} + \Delta G_{-35,j})}}{1 + e^{-(\Delta G_{-10,i} + \Delta G_{-35,j})}} + \epsilon, \qquad TR_{+}(i,j) = \alpha \frac{e^{-(\gamma_{+} + \Delta G_{-10,i} + \Delta G_{-35,j})}}{1 + e^{-(\gamma_{+} + \Delta G_{-10,i} + \Delta G_{-35,j})}} + \epsilon, \tag{7}$$

where ϵ and $\alpha + \epsilon$ are the theoretical minimal and maximal transcription rates, respectively. Both α and ϵ are assumed constant across conditions.

Model fitting with single transcription factor

We were interested in the ability of the model to describe the fold change in expression in the presence of inducer. We thus fit the logarithms of the transcription rates predicted by the model to experimental data. We assumed that the recorded fluorescence is proportional to the transcription rate. Thus in the case of a single transcription factor, we assumed that our measurements under the different conditions, $F_\pm(i,j)$, could be related to the transcription rates as $F_\pm(i,j) = A \cdot TR_\pm(i,j)$, with an unknown proportionality constant A. For example, in the uninduced case we have

$$F_-(i,j) = A\left[\alpha \frac{e^{-(\Delta G_{-10,i}+\Delta G_{-35,j})}}{1+e^{-(\Delta G_{-10,i}+\Delta G_{-35,j})}} + \epsilon\right] = \tilde{\alpha}\frac{e^{-(\Delta G_{-10,i}+\Delta G_{-35,j})}}{1+e^{-(\Delta G_{-10,i}+\Delta G_{-35,j})}} + \tilde{\epsilon},$$

with $\tilde{\alpha} = A\alpha$ and $\tilde{\epsilon} = A\epsilon$.

In the case of a single transcription factor, the transcription rates are given by Eq. (7). We had two sets of data, corresponding to whether arabinose (for AraC), or 3-OC12-HSL (for LasR) was used as inducer. We fit the model to each of these sets of data separately.

As the proportionality constant A between the recorded fluorescence and the transcription rate was unknown, we fit the parameters $\tilde{\alpha}$ and $\tilde{\epsilon}$ directly. We set

$$b_0 = -\Delta G_{-10,1} - \Delta G_{-35,1}, \tag{8}$$

and fit the other parameters relative to this value. For example, we set $-\Delta G_{-10,2} - \Delta G_{-35,3} = b_0 + b_{-10,2} + b_{-35,3}$. Thus we needed to fit 13 parameters b_0, $b_{-10,2}, \ldots, b_{-10,8}$, and $b_{-35,2}, \ldots, b_{-35,6}$. In addition, we needed to fit the parameter γ_+ in the induced state. In total, we thus fit 16 parameters to data. To do so, we minimized

$$\begin{aligned}
&[\log(F_-(1,1)(\tilde{\alpha},\tilde{\epsilon},b_0)) - \log(\mathrm{Data}_{\mathrm{uninduced}}(1,1))]^2 + \\
&\sum_{i=1}^{8}\sum_{j=1}^{6}{}' [\log(F_-(i,j)(\tilde{\alpha},\tilde{\epsilon},b_0,b_{-10,i},b_{-35,j})) - \log(\mathrm{Data}_{\mathrm{uninduced}}(i,j))]^2 + \\
&[\log(F_+(1,1)(\tilde{\alpha},\tilde{\epsilon},b_0,\gamma_+)) - \log(\mathrm{Data}_{\mathrm{induced}}(1,1))]^2 + \\
&\sum_{i=1}^{8}\sum_{j=1}^{6}{}' [\log(F_+(i,j)(\tilde{\alpha},\tilde{\epsilon},b_0,b_{-10,i},b_{-35,j},\gamma_+)) - \log(\mathrm{Data}_{\mathrm{induced}}(i,j))]^2 .
\end{aligned} \tag{9}$$

The primed double sum means that the sum is taken over all pairs i,j except the pair $i=1, j=1$.

We minimized this nonlinear function using *DEoptim* package in R. Other optimization algorithms gave similar results, but those obtained with *DEoptim* gave the most consistent results. We tested convergence by increasing the maximal number of iterations.

We note that we made three measurements of fluorescence for each promoter in the library both in the induced and uninduced states. We fit the model to the average of these three measurements.

Mathematical model with activator and repressor

In the presence of a repressor and activator, the derivation is largely the same, except that we have two additional states: 000, $A00$, $0R0$, $AR0$, 00σ, and $A0\sigma$. We assume that states $0R\sigma$ and $AR\sigma$ have zero probability. We now have the following changes in energies, all of which are again measured relative to the state 000,

$$\Delta G_{A00} = -\log(K_A H^+(I_A)) \tag{10}$$
$$\Delta G_{00\sigma} = \Delta G_{-10,i} + \Delta G_{-35,j} \tag{11}$$
$$\Delta G_{A0\sigma} = \Delta G_{A00} + \Delta G_{00\sigma} + \beta_{A\sigma} \tag{12}$$
$$\Delta G_{0R0} = -\log(K_R H^-(I_R)) \tag{13}$$
$$\Delta G_{AR0} = \Delta G_{A00} + \Delta G_{0R0}. \tag{14}$$

Here H^+ and H^- are increasing and decreasing Hill functions, respectively, of activator inducer concentration I_A and repressor inducer concentration I_R. The probability that the σ-factor is bound to DNA is given by

$$P(\sigma) = \frac{e^{-\Delta G_{00\sigma}} + e^{-\Delta G_{A0\sigma}}}{1 + e^{-\Delta G_{A00}} + e^{-\Delta G_{00\sigma}} + e^{-\Delta G_{A0\sigma}} + e^{-\Delta G_{0R0}} + e^{-\Delta G_{AR0}}}. \quad (15)$$

When we allow for the presence of repressor, the system has four extremal states: activator inducer absent/present and IPTG absent/present. The four states are denoted by $++$, $+-$, $-+$, and $--$, where the first symbol corresponds to activator inducer and the second to IPTG. Here IPTG prevents the binding of repressor, but not completely. Thus we will assume that even in the presence of IPTG, there is some level of repression. This makes the $-+$ state (activator inducer absent, IPTG present) different from the uninduced state of the activator-only system.

State $-/+$. Activator inducer absent, IPTG present. Since activator does not bind to DNA in this state, we assume that all states that include A have zero probability. Eq. (15) reduces to

$$P_{-+}(\sigma) = \frac{e^{-\Delta G_{00\sigma}}}{1 + e^{-\Delta G_{00\sigma}} + \eta}, \quad (16)$$

where $\eta = e^{-\Delta G_{0R0}}$. Proceeding as in the previous section, we introduce the parameter $\gamma_{-+} = \log(1+\eta)$ and multiply the numerator and denominator of Eq. (16) by $(1+\eta)^{-1}$ to obtain

$$P_{-+}(\sigma) = \frac{e^{-(\gamma_{-+} + \Delta G_{-10,i} + \Delta G_{-35,j})}}{1 + e^{-(\gamma_{-+} + \Delta G_{-10,i} + \Delta G_{-35,j})}}. \quad (17)$$

State $+/+$. Activator inducer present, IPTG present. This state corresponds to the induced state of the activator-only system. Assuming again that the probability of the state 00σ is much lower than that of the state $A0\sigma$, we have

$$P_{++}(\sigma) \approx \frac{e^{-\Delta G_{A0\sigma}}}{1 + e^{-\Delta G_{A00}} + e^{-\Delta G_{A0\sigma}} + \eta},$$

where now $\eta = e^{-\Delta G_{0R0}} + e^{-\Delta G_{AR0}}$. If we set $\gamma = \log(1 + e^{-\Delta G_{A00}} + \eta)$, we obtain

$$\begin{aligned} P_{++}(\sigma) &\approx \frac{e^{-(\gamma + \Delta G_{A00} + \Delta G_{-10,i} + \Delta G_{-35,j} + \beta_{A\sigma})}}{1 + e^{-(\gamma + \Delta G_{A00} + \Delta G_{-10,i} + \Delta G_{-35,j} + \beta_{A\sigma})}} \\ &= \frac{e^{-(\gamma_{++} + \Delta G_{-10,i} + \Delta G_{-35,j})}}{1 + e^{-(\gamma_{++} + \Delta G_{-10,i} + \Delta G_{-35,j})}}, \end{aligned} \quad (18)$$

where $\gamma_{++} = \gamma + \Delta G_{A00} + \beta_{A\sigma}$.

State $-/-$. Both activator inducer and IPTG absent. Assuming all states including A have zero probability, we have

$$P_{--}(\sigma) = \frac{e^{-\Delta G_{00\sigma}}}{1 + e^{-\Delta G_{00\sigma}} + e^{-\Delta G_{0R0}}}.$$

Defining $\gamma_{--} = \log(1 + e^{-\Delta G_{0R0}})$ and arguing as before, we have

$$P_{--}(\sigma) = \frac{e^{-(\gamma_{--} + \Delta G_{-10,i} + \Delta G_{-35,j})}}{1 + e^{-(\gamma_{--} + \Delta G_{-10,i} + \Delta G_{-35,j})}}. \quad (19)$$

State $+/-$. Activator inducer present, IPTG absent. Assuming the state $A0\sigma$ is significantly more probable than 00σ, we have

$$P_{+-}(\sigma) \approx \frac{e^{-\Delta G_{A0\sigma}}}{1 + e^{-\Delta G_{A00}} + e^{-\Delta G_{A0\sigma}} + e^{-\Delta G_{0R0}} + e^{-\Delta G_{AR0}}}.$$

With $\gamma = \log(1 + e^{-\Delta G_{A00}} + e^{-\Delta G_{0R0}} + e^{-\Delta G_{AR0}})$, we obtain

$$P_{+-}(\sigma) \approx \frac{e^{-(\gamma+\Delta G_{A0\sigma})}}{1 + e^{-(\gamma+\Delta G_{A0\sigma})}} = \frac{e^{-(\gamma_{+-}+\Delta G_{-10,i}+\Delta G_{-35,j})}}{1 + e^{-(\gamma_{+-}+\Delta G_{-10,i}+\Delta G_{-35,j})}}, \quad (20)$$

where $\gamma_{+-} = \gamma + \Delta G_{A00} + \beta_{A\sigma}$.

Note that Eqs. (17–20) all have the same form and differ only in the constants $\gamma_{\pm\pm}$.

As above in Eq. (7), we assume that the transcription rate is proportional to the binding probability, where ϵ and $\alpha + \epsilon$ are the theoretical minimal and maximal transcription rates, respectively. At full IPTG induction, we therefore fit the data assuming transcription rates

$$TR_{-+}(i,j) = \alpha \frac{e^{-(\gamma_{-+}+\Delta G_{-10,i}+\Delta G_{-35,j})}}{1 + e^{-(\gamma_{-+}+\Delta G_{-10,i}+\Delta G_{-35,j})}} + \epsilon, \qquad TR_{++}(i,j) = \alpha \frac{e^{-(\gamma_{++}+\Delta G_{-10,i}+\Delta G_{-35,j})}}{1 + e^{-(\gamma_{++}+\Delta G_{-10,i}+\Delta G_{-35,j})}} + \epsilon. \quad (21)$$

However, we made one important additional assumption when we fit these models to the data: We allowed for the possibility that repression may not be full. This could happen if there is insufficient repressor for all copies of a promoter within a cell, for example. Thus we assumed that

$$TR_{--}(i,j) = \alpha \left[p \frac{e^{-(\gamma_{--}+\Delta G_{-10,i}+\Delta G_{-35,j})}}{1 + e^{-(\gamma_{--}+\Delta G_{-10,i}+\Delta G_{-35,j})}} + (1-p) \frac{e^{-(\gamma_{-+}+\Delta G_{-10,i}+\Delta G_{-35,j})}}{1 + e^{-(\gamma_{-+}+\Delta G_{-10,i}+\Delta G_{-35,j})}} \right] + \epsilon \quad (22)$$

$$TR_{+-}(i,j) = \alpha \left[p \frac{e^{-(\gamma_{+-}+\Delta G_{-10,i}+\Delta G_{-35,j})}}{1 + e^{-(\gamma_{+-}+\Delta G_{-10,i}+\Delta G_{-35,j})}} + (1-p) \frac{e^{-(\gamma_{++}+\Delta G_{-10,i}+\Delta G_{-35,j})}}{1 + e^{-(\gamma_{++}+\Delta G_{-10,i}+\Delta G_{-35,j})}} \right] + \epsilon. \quad (23)$$

The probabilities p are high in all fits (above 0.95), indicating that repression is nearly complete. However, even a small probability of an unrepressed promoter can have a large effect when the promoter is strong.

Model fitting with activator and repressor

As in the case of a single transcription factor, we were interested in the ability of the model to describe the fold change in expression in the presence of activator inducer and IPTG. We again fit the logarithms of the transcription rates predicted by the model to experimental data. As before, we assumed that our fluorescence measurements were proportional to transcription rates under all conditions, so that $F_{\pm\pm}(i,j) = A \cdot TR_{\pm\pm}(i,j)$ with an unknown proportionality constant A.

Note that if $p = 1$, the four conditions discussed in the previous section differ only in the four coefficients $\gamma_{\pm\pm}$, as we assumed in our model that all other coefficients are unaffected by the presence of activator inducer or IPTG. The model included unknown theoretical minimal and maximal transcription rates ϵ and $\alpha + \epsilon$, respectively. As the proportionality constant A between the recorded fluorescence and the transcription rate was unknown, we again fit the parameters $\tilde{\alpha} = A\alpha$ and $\tilde{\epsilon} = A\epsilon$, as in the single transcription factor case.

To reduce the number of parameters, we first defined the reference parameter

$$b_4 = -(\gamma_{++} + \Delta G_{-10,1} + \Delta G_{-35,1}),$$

following the same reasoning that lead to the reference energy parameter defined in Eq. (8). We then defined other parameters relative to b_4 as

$$b_1 + b_4 = -(\gamma_{--} + \Delta G_{-10,1} + \Delta G_{-35,1}),$$
$$b_2 + b_4 = -(\gamma_{+-} + \Delta G_{-10,1} + \Delta G_{-35,1}),$$
$$b_3 + b_4 = -(\gamma_{-+} + \Delta G_{-10,1} + \Delta G_{-35,1}).$$

The parameters $b_{-10,i}$ and $b_{-35,j}$ were now measured relative to the b_4 baseline as well. For example, the parameter $b_{-35,2}$ satisfies

$$b_1 + b_4 + b_{-35,2} = -(\gamma_{--} + \Delta G_{-10,1} + \Delta G_{-35,2}),$$

giving

$$b_{-35,2} = -(\gamma_{--} + \Delta G_{-10,1} + \Delta G_{-35,2}) - b_1 - b_4$$
$$= -(\Delta G_{-35,2} - \Delta G_{-35,1}).$$

Using a reference parameter allowed us to reduce the total number of parameters to 19.

To fit the model, we minimized the equivalent of the cost function given in Eq. (9) (the only difference is that we now have the two repressed states). We again minimized this nonlinear function using the*DEoptim* package in R.

Commented code, as well as all collected data are available in the Github repository at `https://github.com/josic/Promoter-Engineering`
