# Supplementary material for: Tuning the dynamic range of bacterial promoters regulated by ligand-inducible transcription factors
Source: Nat Commun. 2018 Jan 4;9:64. doi: 10.1038/s41467-017-02473-5 (PMC5754348; doi:10.1038/s41467-017-02473-5)
Supplement: Supplementary file 3 — Description of Additional Supplementary Files [file 41467_2017_2473_MOESM3_ESM.pdf]

Description of Additional Supplementary Files

File Name: Supplementary Data 1
Description: Promoter sequences that list the operator order, -35 and -10 sequences, and activator binding site sequences.

File Name: Supplementary Data 2
Description: Combinatorial promoter library with array of -35 and -10 sequences.

File Name: Supplementary Data 3
Description: Combinatorial library data for AraC- as well as LasR-regulated one-input promoters tested in strain CY015.

File Name: Supplementary Data 4
Description: Induction data for AraC- as well as LasR-regulated one-input promoters with diverse dynamic ranges.

File Name: Supplementary Data 5
Description: Combinatorial library data for AraC- as well as LasR-regulated multiinput (LacI) hybrid promoters tested in strain CY021.

File Name: Supplementary Data 6
Description: Sequences of multi-input hybrid promoters controlled by diverse transcription factors.

File Name: Supplementary Data 7
Description: Induction data for two-input hybrid promoters controlled by diverse transcription factors.

File Name: Supplementary Data 8
Description: Induction data for three-input hybrid promoters controlled by diverse transcription factors.
